# Quantitative System Pharmacology Analysis of Oral Valiltramiprosate/ALZ‐801 Predicts Slowing of Alzheimer’s Disease Progression by Anti‐Amyloid Oligomer and APOE4 Structural Corrector Modes of Action

**DOI:** 10.1002/alz70861_108561

**Published:** 2025-12-23

**Authors:** Jean Schaefer, Hugo Geerts, Shaina M Short, Jeremy Yu, Susan Abushakra, Aidan Power, Martin Tolar, John A. Hey

**Affiliations:** ^1^ Alzheon, Framingham, MA USA; ^2^ Certara Predictive Technologies, Berwyn, PA USA; ^3^ Certara Predictive Technologies, Park City, UT USA; ^4^ Alzheon, Inc., Framingham, MA USA

## Abstract

**Background:**

APOE4 genotype is a major risk factor in late‐onset Alzheimer’s disease (AD), with APOE4/4 homozygous patients showing the highest risk and most aggressive progression. Valiltramiprosate is an inhibitor of β‐amyloid (Aβ) oligomer formation in development as a disease‐modifying AD treatment. It has been reported that valiltramiprosate, via its active molecules tramiprosate and 3‐SPA, also exhibits APOE4 structure corrector activities, converting the abnormal APOE4 protein to the more physiological APOE3‐like conformation and function (Nemergut et al., 2023). The present quantitative system pharmacology (QSP) analysis aims to determine the effects of valiltramiprosate on AD progression by integrating its anti‐oligomer and APOE4 corrector modes of action (MOA).

**Method:**

We developed novel QSP trajectory models for the Aβ aggregation cascade and hippocampal volume changes in AD by APOE4 genotype. The valiltramiprosate pharmacology was implemented based on its anti‐Aβ oligomer and APOE4 corrector MOAs. We projected brain drug exposures based on clinical pharmacokinetic data of valiltramiprosate 265 mg BID and the preclinical brain/plasma relationship. The effects of valiltramiprosate on Aβ forms (monomer, oligomer, protofibril, and plaque) and hippocampal volume over a simulated 2‐year treatment in AD were examined.

**Result:**

The QSP analysis shows that valiltramiprosate treatment substantially reduces Aβ oligomer formation and hippocampal atrophy in all APOE genotypes in an exposure‐dependent manner. The combined anti‐oligomer + APOE4 corrector actions produce an additive, positive impact on inhibiting Aβ oligomer and protofibril formation (10‐20%), and a larger effect on reduction of hippocampal atrophy especially in APOE4/4 subjects. Sensitivity analyses for the two MOAs suggest that the effect is driven primarily by the anti‐Aβ oligomer pharmacology, with a smaller contribution from the APOE4 corrector mechanism operative in APOE4 carrier AD patients.

**Conclusion:**

The results suggest that the primary molecular MOA driver of valiltramiprosate to reduce AD neurodegenerative pathology is mediated by its anti‐aggregation action, which in turn prevents formation of soluble neurotoxic oligomers and protofibrils without directly reducing amyloid plaques load. The APOE4 corrector MOA of valiltramiprosate may partially contribute to its clinical efficacy in APOE4/4 and APOE4/3 AD patients by switching the phenotype to APOE3/3 phenotype.

